# DEALING WITH INJURED OR REPAIRED ANKLES IN OLDER ADULTS: ASSESSING ANKLE IMMOBILIZATION METHODS DURING LOCOMOTION

**DOI:** 10.1093/geroni/igad104.2454

**Published:** 2023-12-21

**Authors:** Clark Dickin

**Affiliations:** Ball State University, Muncie, Indiana, United States

## Abstract

Orthopedic walking boots (OWB) are an alternative to casting and immobilizing an injured ankle. Unfortunately, OWB causes a change in leg-length and pelvis angle which alters gait and postural stability and can lead to chronic back pain. Immobilizing the ankle without changing leg-length would be beneficial, especially for individuals with impaired gait and postural stability. The purpose of this study was to assess level-ground walking and stair negotiation in healthy older adults across three ankle support conditions (1) OWB, (2) TayCo ankle brace (TAB) and (3) athletic footwear (SHOD). Sixteen older adults (69+/-7.0 years) performed 2-3 trials of walking, stair ascent, and stair descent using each of the three ankle support conditions. Ankle motion in all three planes was monitored using 3D motion capture cameras to assess differences during walking and stair negotiation. For all tasks, significant differences were revealed between the SHOD and both ankle support methods for all three planes (p < .001). Significant differences were also revealed between OWB and TAB during walking in the transverse plane (abd/adduction) (p = .036 OWB>TAB) and for stair descent in the sagittal plane (dorsi/plantarflexion) (p = .001 TAB>OWB). No other differences were observed between the OWB and TAB for walking or stair negotiation. Ankle motion was similarly controlled with both the OWB and TAB indicating that either method would be appropriate to immobilize an injured ankle, however the TAB does not alter leg length or pelvic tilt since it fits over the individual’s footwear.

